# Isolation and identification of antagonistic fungi for biocontrol of *Impatiens hawkeri* leaf spot disease and their growth-promoting potential

**DOI:** 10.3389/fmicb.2025.1584353

**Published:** 2025-05-16

**Authors:** Huali Li, Mingguo Yang, Jian Liu, Yajiao Sun, Huajun Yang, Junjia Lu

**Affiliations:** ^1^College of Landscape Architecture and Horticulture Sciences, Southwest Forestry University Sciences, Kunming, China; ^2^Yunnan Key Laboratory of Forest Disaster Warning and Control, Southwest Forestry University, Kunming, China; ^3^Zhaoyang District Comprehensive Administrative Law Enforcement Bureau of Zhaotong City, Zhaotong, Yunnan, China

**Keywords:** *Impatiens hawkeri*, leaf spot, *Fusarium solani*, biological control, mechanisms of inhibiting pathogenic fungi, growth-promoting function

## Abstract

*Impatiens hawkeri*, a plant of great horticultural significance with high ornamental and economic value, is frequently afflicted by leaf spot disease caused by *Stagonosporopsis cucurbitacearum*, and effective biological control methods for this disease are yet to be fully explored. To address this issue, the plate confrontation method was used to isolate five antagonistic fungal strains from *Quercus spinosa*, which showed strong inhibitory effects against *S. cucurbitacearum* with inhibition rates of 53.12% for CG6, 43.42% for CG7, 68.13% for CY12, 54.30% for CJ18, and 67.23% for CJ19, respectively. Among them, strain CY12 demonstrated the highest antifungal activity, significantly suppressing the mycelial growth of *S. cucurbitacearum*. Through morphological observation and sequence analyses of ITS, TEF, and RPB2, CY12 was identified as *Fusarium solani*. Further experiments revealed that CY12 could produce cell wall hydrolases and exhibited multiple growth-promoting properties, such as phosphorus and potassium solubilization, nitrogen fixation, and indoleacetic acid (IAA) production, and it also produced various cell wall-degrading enzymes like amylase, cellulase, and *β*-glucanase. Additionally, CY12 showed strong antagonistic effects against six common ornamental plant pathogens, with an inhibition rate of 71.41% against the pathogen causing Rhododendron leaf spot disease. Pot experiments indicated that CY12 effectively reduced the occurrence of leaf spot disease on *I. hawkeri* plants, increased the seed germination rate from 93% in the control group to 98.33%, and promoted seedling growth. These results suggest that the endophytic fungus CY12 has strong antifungal and growth-promoting properties, providing potential strain resources for the biological control of leaf spot disease in *I. hawkeri* and the development of fungal fertilizers, and further exploration of its field application effects and action mechanisms is necessary.

## 1 Introduction

Leaf spot disease is a common plant pathology that affects a wide range of species, including ornamental plants and economically valuable crops. Once infected, plants develop spots of varying sizes, typically round or irregular in shape, on their leaves. These lesions are primarily yellow-brown in color (Matic et al., 2020). As the disease progresses, the spots darken to brown or even black, expanding and merging with adjacent lesions (Park et al., 2024). In severe cases, leaf spot disease can cause extensive tissue necrosis, leading to leaf wilting, defoliation, and plant decline.

The leaf spot disease of *I. hawkeri* is particularly prevalent and causes significant damage. This will not only lead to a decline in the quality of *I. hawkeri*, but also reduce the yield, thus decreasing its market competitiveness. In addition, the prevention and control costs will also increase. (Simko et al., 2023). Under conditions of warmth, high humidity, and poor ventilation, factors that facilitate pathogen proliferation, disease-causing agents multiply and spread more efficiently (Liu et al., 2020). This often results in widespread infections that can ultimately kill the entire plant, severely diminishing the ornamental value of *I. hawkeri* (Wang Y. et al., 2024).

Currently, various chemical fungicides, such as carbendazim and thiophanate-methyl, are available for managing leaf spot diseases. Additionally, compounds like mancozeb, 2,1-benzisoxazole (Park et al., 2024), and propiconazole (Imran et al., 2020) have demonstrated inhibitory effects against certain leaf spot pathogens. However, the application of chemical fungicides is often associated with environmental pollution and the emergence of fungicide-resistant pathogen strains (Bhatnagar et al., 2024).

One alternative to chemical pesticides is the use of microbial control agents. Endophytic fungi, a unique biological resource within plants, have attracted increasing attention due to their ability to colonize plant tissues and influence plant growth and reproduction. For example, Németh et al. (2023) found in their research the strain *Ampelomyces* can serve as a biological control fungus for powdery mildew and can be used to combat various powdery mildew pathogens. Ma et al. (2023) discovered that the endophytic fungus *Phomopsis liquidambaris* can inhibit peanut root rot caused by *Fusarium oxysporum*. These fungi contribute to host plants’ resistance against both biotic and abiotic stresses and serve as sources of bioactive compounds (Yang et al., 2023). Plant endophytes have gained recognition for their non-pathogenic nature, non-toxicity (Gupta et al., 2020), and reduced likelihood of inducing resistance in plants. Additionally, they are environmentally friendly, pollution-free, and safe for humans and livestock (Thambugala et al., 2020; Wei et al., 2023). Biocontrol fungi suppress plant pathogens through various mechanisms. They produce metabolites such as antibiotics, volatile compounds, and enzymes that inhibit the growth of pathogenic fungi. They also compete with pathogens for space, carbon, nitrogen, and mineral resources, engage in parasitism, and trigger systemic resistance in plants, thereby reducing pathogen activity (Yan et al., 2019). Some plant endophytes produce cell wall-degrading enzymes, including proteases and *β*-glucanases (Baron and Rigobelo, 2022), and strengthen plant disease resistance by regulating the plant immune system or directly synthesizing bioactive compounds that inhibit pathogens (Qin et al., 2024). Moreover, certain plant endophytes exhibit growth-promoting traits such as phosphorus solubilization and potassium release (Kharkwal et al., 2024). They can also synthesize hormones like indole-3-acetic acid (IAA), which influence plant growth and development, ultimately promoting host plant growth.

*Quercus spinosa* is an evergreen tree or shrub known for its exceptional tolerance to poor soils, cold, and drought conditions. It typically thrives at altitudes between 900 and 3,000 meters (Chen and Huang, 1998) and is frequently found growing on exposed rocky cliffs (Ju et al., 2019). Seminal work by Chen et al. (2021) established thatalpine plants may harbor more diverse endophytic fungi compared to common horticultural plants. Due to their long-term growth in harsh environments, endophytic fungi have evolved resistance mechanisms adapted to their host plants to survive within them.

In this study, fungi with strong antagonistic effects against the leaf spot disease of *I. hawkeri* were isolated and screened from the healthy tissues of *Q. spinosa*. By assessing their antifungal activity, enzyme production, and growth-promoting properties, a dominant strain was identified. Morphological and molecular analyses were then performed to classify this strain, followed by an investigation of its antifungal spectrum. Finally, pot experiments were conducted to evaluate its effectiveness in enhancing disease resistance and promoting the growth of *I. hawkeri*. Previous studies mainly focused on using bacterial biocontrol agents and mycoviruses to combat plant leaf spot diseases (Ahsan et al., 2022; Liu et al., 2022). In this study, endophytic fungi were isolated from *Q. spinosa* to combat the leaf spot disease of *I. hawkeri*. This discovery of brand – new biological resources helps to further clarify the functions of plant endophytic fungi in the ecosystem and deepen the research on the interaction mechanism between plants and microorganisms. This research aims to explore the potential of endophytic fungi in strengthening plant disease resistance, reducing dependence on chemical pesticides, lowering production costs, and decreasing environmental pollution. The findings are expected to provide valuable insights for the development of biological control agents and bio-fertilizers, contributing to the advancement of sustainable agriculture.

## 2 Materials and methods

### 2.1 Materials

In October 2023, *Q. spinosa* samples were collected from Daguniushan Mountain, Dongchuan, Kunming, Yunnan Province (altitude 4017.3 m). Healthy roots, stems, and leaves were carefully collected, immediately sealed in sterile bags, and placed in an ice-filled container for transport. The samples were then stored in the laboratory of the College of Landscape Architecture and Horticulture Sciences at Southwest Forestry University.

*Impatiens hawkeri* plants were purchased from Jiangsu Flower Breeding Base Co., Ltd. in October 2023 and cultivated in the laboratory for six months before use in the experiments. *I. hawkeri* seeds were obtained from Mozhichun Nursery Stock Management Department in Muyang County, Jiangsu Province.

The pathogen strains used in this study, including *Stagonosporopsis cucurbitacearum* (the causal agent of *I. hawkeri* leaf spot disease), *Fusarium oxysporum* (responsible for *I. hawkeri* wilt disease), *Pestalotiopsis* (causing Rhododendron leaf spot disease), *Fusarium sambucinum* (associated with *Hibiscus mutabilis* leaf spot disease), *Apiospora intestini* (triggering *Hedera helix* leaf blight disease), *Epicoccum sorghinum* (linked to Hydrangea leaf spot disease), and *Botrytis cinerea* (leading to Blueberry gray mold disease), were all maintained and stored in the laboratory of the College of Landscape Architecture and Horticulture Sciences, Southwest Forestry University. The culture medium formulations used are shown in Table 6.

### 2.2 Isolation of antagonistic fungi and screening of dominant strains

Endophytic fungi were isolated using the tissue isolation method (Kuo et al., 2021). The roots, stems, and leaves of *Q. spinosa* were thoroughly washed under running tap water to remove surface contaminants before being transferred to an ultra-clean workbench for disinfection. The disinfection procedure involved sequential treatments with 75% ethanol, 1% sodium hypochlorite, and sterile water. Specifically, plant tissues were soaked in 75% ethanol (2 min for roots, 1 min for stems and leaves), rinsed once with sterile water, then soaked in 1% sodium hypochlorite (3 min for roots, 2 min for stems and leaves), followed by 3 to 5 rinses with sterile water. After disinfection, the samples were cut into 1 cm^2^ pieces using a sterile scalpel and placed on Potato Dextrose Agar (PDA) medium plates. To verify the effectiveness of the disinfection process, sterile water from the final leaf rinse was spread onto a separate PDA plate as a control; if no colonies formed, the disinfection was considered successful. Plates were incubated upside down in a constant-temperature incubator at 28°C for 72 h. Colony growth was observed, and single colonies with distinct morphologies, colors, and growth patterns were selected for purification and further cultivation. The fully purified strains were numbered systematically and stored at 4°C for future use.

For strain screening, the plate confrontation method (Putri et al., 2022) was used. A 5-mm fungal punch was used to extract a plug from the pathogen responsible for *I. hawkeri* leaf spot disease after 7 days of culture. This plug was then inoculated at the center of a PDA plate using a sterile inoculating needle. The antagonistic fungi were subsequently inoculated 2 cm away in a cross pattern. Plates were incubated at 30°C for 5 to 7 days, after which the inhibition zone and its diameter were observed and recorded. Strains exhibiting strong antifungal activity were selected for further study.

$\begin{array}{l} \text{Theantifungalrate}\;(\%)=(\begin{array}{l} \text{the diameter of the control colony}\\ –\text{the diameter of the treated colony} \end{array})\\ /\text{the diameter of the control colony}\times 100 \end{array}$

### 2.3 Detection of enzyme production by antagonistic fungi on plates

#### 2.3.1 Plate detection of amylase, cellulase, and pectinase production

A 6-mm hole puncher was used to extract plugs from the edges of the dominant biocontrol strain colonies. These plugs were then inoculated at the center of specific detection media for amylase, cellulase, and pectinase production, respectively (Pereyra et al., 2020). Each experiment was conducted in triplicate, with plates incubated upside down at 30°C for 7 days. After the incubation period, 1% iodine solution was added to the amylase detection plate and left for 30 min to develop color. The iodine solution was then removed, and the plate was examined for the presence of a decolorized zone. For cellulase detection, a 1 mg/mL Congo red staining solution was added to the plate. After staining for 1 h, the Congo red solution was discarded, and 1 mol/L sodium chloride solution was added for a 30-min elution. The eluent was then removed, and the plate was inspected for the formation of a decolorized zone. Similarly, for pectinase detection, 1% iodine solution was added and left for 30 min for staining. The staining solution was then removed, followed by the addition of 1 mol/L sodium chloride solution for a 1-h elution. The eluent was discarded, and the presence of a decolorized zone around the colonies was observed.

#### 2.3.2 Plate detection of *β*-glucanase and protease production

A 6-mm hole puncher was used to extract plugs from the edges of biocontrol strain colonies that had been cultured for over 7 days and exhibited healthy growth. These fungal plugs were then inoculated onto β-glucanase screening medium and protease detection medium (Zhou et al., 2022). Each experiment was performed in triplicate, with plates incubated upside down at 30°C for 7 days. After incubation, the plates were examined for the formation of transparent zones, and the results were recorded.

### 2.4 Determination of the growth-promoting abilities of antagonistic fungi

#### 2.4.1 Qualitative plate detection of phosphate-solubilizing, potassium-releasing and siderophore-producing abilities

The selected biocontrol fungi with healthy growth were inoculated onto organic phosphorus, potassium-solubilizing, and CAS media. Each strain was tested in triplicate and incubated upside down in a constant-temperature incubator at 28°C. After 5 days, the presence of clear or halo zones was observed (Akasah and Chou, 2023; Doilom et al., 2020; Ozimek and Hanaka, 2020). The diameter (*D1*) of the transparent or halo zones and the colony diameter (*d1*) were measured. The ratio *D1/d1* was used to assess phosphate-solubilizing, potassium-releasing and Siderophore-Producing abilities, with larger ratios indicating stronger capabilities. If there is no transparent circle or halo, it indicates that the strain does not possess phosphate – solubilizing, potassium – releasing and siderophore – producing abilities.

#### 2.4.2 Qualitative plate detection of nitrogen-fixing ability

Biocontrol fungi were inoculated onto nitrogen-free medium, with each strain tested in triplicate. The inoculated plates were incubated upside down in a constant-temperature incubator at 28°C for 7 days. After five consecutive subcultures, if the strain continued to form normal colonies, it was considered to possess nitrogen-fixing ability (Yang et al., 2014). If the strain fails to grow normally, it is considered that the strain does not have nitrogen – fixing ability.

#### 2.4.3 Determination of the ability to secrete indole-3-acetic acid (IAA)

##### 2.4.3.1 Qualitative analysis

The Salkowski colorimetric method was used (Imran et al., 2021). The selected strains were inoculated into King’s B culture medium with and without tryptophan and subjected to liquid fermentation in a 28°C constant-temperature shaker for 7 days. Each experiment was repeated three times. After fermentation, 5 mL of the broth was centrifuged, and 2 mL of the supernatant was mixed with an equal volume of ferric chloride colorimetric solution. The mixture was left to stand in the dark for 30 min, after which color changes were observed.

##### 2.4.3.2 Quantitative analysis

A series of indole-3-acetic acid (IAA) standard solutions with varying concentrations were prepared. Using the same color development method as described above, absorbance at 530 nm was measured with an ultraviolet spectrophotometer. A standard curve was plotted, and an equation was derived. The OD values of color-producing strains from the qualitative analysis were measured at 530 nm and substituted into the equation to determine the amount of IAA produced.

### 2.5 Molecular biology and morphological identification of strain CY12

#### 2.5.1 Molecular biology identification

DNA of strain CY12 was extracted following the instructions of a fungal genome extraction kit. Universal primers ITS1 and ITS4, along with translation elongation factor primers EF728F and EF986R, and gene primers RBP2-5F2 and RBP2-11aR, were used for PCR amplification of the genomic DNA (Claerbout et al., 2023; Esmaeili Taheri et al., 2017). Primer details are listed in Table 1. All primers were synthesized by Shanghai Sangon Biotech Co., Ltd. The following are the PCR amplification programs for these several pairs of primers: ITS1 and ITS4 primers: Pre-denaturation at 94°C for 5 min; Denaturation at 94°C for 30 s, annealing at 55°C for 30 s, extension at 72°C for 1 min, a total of 35 cycles; Final extension at 72°C for 10 min. EF728F and EF986R primers: Usually, the program adopted is pre-denaturation at 94°C for 5 min; Then denaturation at 94°C for 30 s, annealing at 55°C for 30 s, extension at 72°C for 1 min, and 34 cycles are carried out; Finally, extension at 72°C for 10 min. RBP2 – 5F2 and RBP – 11aR primers: Generally, pre-denaturation at 94°C for 3 min; Then denaturation at 94°C for 30 s, annealing at 58°C for 30 s, extension at 72°C for 2 min, with 32 cycles; Finally, extension at 72°C for 10 min. The amplified PCR products were sent to the same company for sequencing. Sanger sequencing results were aligned and translated using the NCBI database. A phylogenetic tree was constructed with MEGA 11.0 software using the maximum likelihood method, and its reliability was assessed with 1,000 bootstrap replicates (Bockus et al., 2010; O’Donnell et al., 2022) to accurately determine the species of the endophytic fungus.

**Table 1:** Primer sets and corresponding amplification targets.

Locus	Gene product	Primer	Sequence (5′-3′)
ITS	Internal transcribed spacer primer	ITS1	TCCGTAGGTGAACCTGCGG
		ITS4	TCCTCCGCTTGATATGC
TEF	Translation elongation factor 1-o.	EF728F	CATCGAGAAGTTCGAGAAGG
		EF986R	TACTTGAAGGAACCCTTACC
RPB2	RNA polymerase second largest subunit	5F2	GGGGWGAYCAGAAGAAGGC
		11aR	GCRTGGATCTTRTCRTCSACC

#### 2.5.2 Morphological identification

Strain CY12 was inoculated onto PDA plates and incubated at 28°C for 7 days. Fungal morphology was observed and recorded throughout the incubation period. An inoculation needle was used to gently scrape a small amount of mycelium from normally grown colonies. The mycelium was mixed with sterile water, and an appropriate amount was placed on a glass slide for microscopic observation of its morphology under an optical microscope (Wang et al., 2020). To observe fungal spores using the slide culture method, a 1-mm thick slice of Czapek’s medium was cut and placed on a slide. A sterilized inoculation needle was used to transfer mycelium from the strain onto one side of the medium. A cover slip was placed over it to create a wet chamber slide culture. The conidia and conidiogenous cells were observed at regular intervals, and micrographs were taken to document their structures.

### 2.6 Determination of the antifungal spectrum of CY12

A confrontation culture experiment was conducted between the biocontrol strain CY12 and various plant pathogenic fungi preserved in the laboratory. Using the five – point confrontation assay, the pathogen was inoculated at the center of the antagonistic fungus for co-cultivation. Each experiment was performed in triplicate, with Petri dishes incubated upside down at 28°C for 5 days (Gorai et al., 2021). Throughout the incubation period, colony diameters were measured, and inhibition rates were calculated to assess the antifungal activity of CY12.

### 2.7 *In vivo* antifungal experiment of CY12 against leaf spot disease of potted *Impatiens hawkeri*

A conidia suspension of *Stagonosporopsis cucurbitacearum* was prepared and adjusted to a concentration of 1 × 10^8^ spores/mL. Additionally, a fermentation broth of the CY12 strain was prepared. Healthy *I. hawkeri* plants that had been cultivated for 9 months were selected. On each plant, two branches with comparable growth vigor were chosen. Using a sterile scalpel, small wounds were created on the leaves. On one branch, 2 mL of the pathogen conidia suspension was applied directly to the wounds. On the second branch, 2 mL of the pathogen conidia suspension broth was first applied, and after 4 h, an additional 2 mL of CY12 fermentation broth was introduced (Fan et al., 2021). Each experimental group was replicated three times to ensure data reliability. The plants were then transferred to an indoor environment maintained at a constant temperature of 25°C and incubated for 7 days. During this period, observations were made, and any changes in disease progression or fungal interaction were recorded.

### 2.8 Growth-promoting tests of CY12 on seed germination and seedling roots of *Impatiens hawkeri*

The supernatant of the CY12 fermentation broth was collected for later use. A total of 400 *I. hawkeri* seeds, uniform in size, plump, and with intact outer coverings, were selected. After undergoing appropriate disinfection procedures, the seeds were divided into two equal batches of 200 each. One batch was immersed in the CY12 fermentation supernatant, while the other was placed in Potato Dextrose Broth (PDB) medium as a control. Both treatments were incubated under continuous oscillation at 200 r/min at 37°C for 8 h. Once removed, the treated seeds were used for seed germination and growth-promoting experiments (Daigham et al., 2023; Parveen et al., 2019).

The two batches of treated seeds were further divided into groups of 50 seeds each and placed in Petri dishes lined with two layers of filter paper. A small amount of sterile water was added to keep the filter paper moist. The Petri dishes were then sealed with plastic wrap, with small air holes made for proper ventilation. Each treatment was replicated three times and incubated at a constant temperature of 25°C with 80% relative humidity in darkness. Germination progress was recorded every other day, and the seed germination rate was calculated based on the number of seeds that successfully germinated.

One day after the two groups of seeds, control and CY12 treatment, reached their peak germination rates, they were transplanted into pots containing nutrient-rich soil. The plants were watered regularly in appropriate amounts, and their germination and growth trends were observed. The number of germinated seeds was counted, root and shoot lengths were measured, and the final germination rate was determined.

$\text{Germination rate}(\%)=(\begin{array}{l} \text{Number of germinated seeds}\\ /\text{Total number of tested seeds} \end{array})\times 100$

### 2.9 Data analysis

The data were conducted with statistical software Excel 2016 and SPSS 22.0. One-way ANOVA was used to evaluate the statistical significance of each sample (*p* < 0.05), and then Duncan’s multi-range test was carried out. Data were visualized using AdobePhotoshop 2023 and Adobe Illustrator 2020.

## 3 Results and analysis

### 3.1 The isolation and screening of superior strains from biocontrol fungi

A total of 53 endophytic fungi were isolated and purified from the tissues of *Q. spinosa*, with 21 strains obtained from roots, 19 from stems, and 13 from leaves. The strains isolated from the roots are named starting with “CG,” those isolated from the stems are named starting with “CJ,” and the strains isolated from the leaves are named starting with “CY.” These strains were subjected to a plate inhibition assay against the pathogen responsible for *I. hawkeri* leaf spot disease. Among them, nine strains exhibited strong inhibitory effects. After eliminating four slow-growing strains, five dominant strains were selected: two from roots, two from stems, and one from leaves, designated as CG6, CG7, CJ18, CJ19, and CY12. The five selected strains underwent a five-point confrontation assay against the more virulent pathogen strain responsible for *I. hawkeri* leaf spot disease. The inhibition rates of CG6, CG7, CJ18, CJ19, and CY12 were 53.12, 43.42, 67.23, 54.31, and 68.13%, respectively. Among them, CY12 demonstrated the highest inhibition rate (68.13%), while CG7 exhibited the lowest (43.42%).

**Figure 1:**
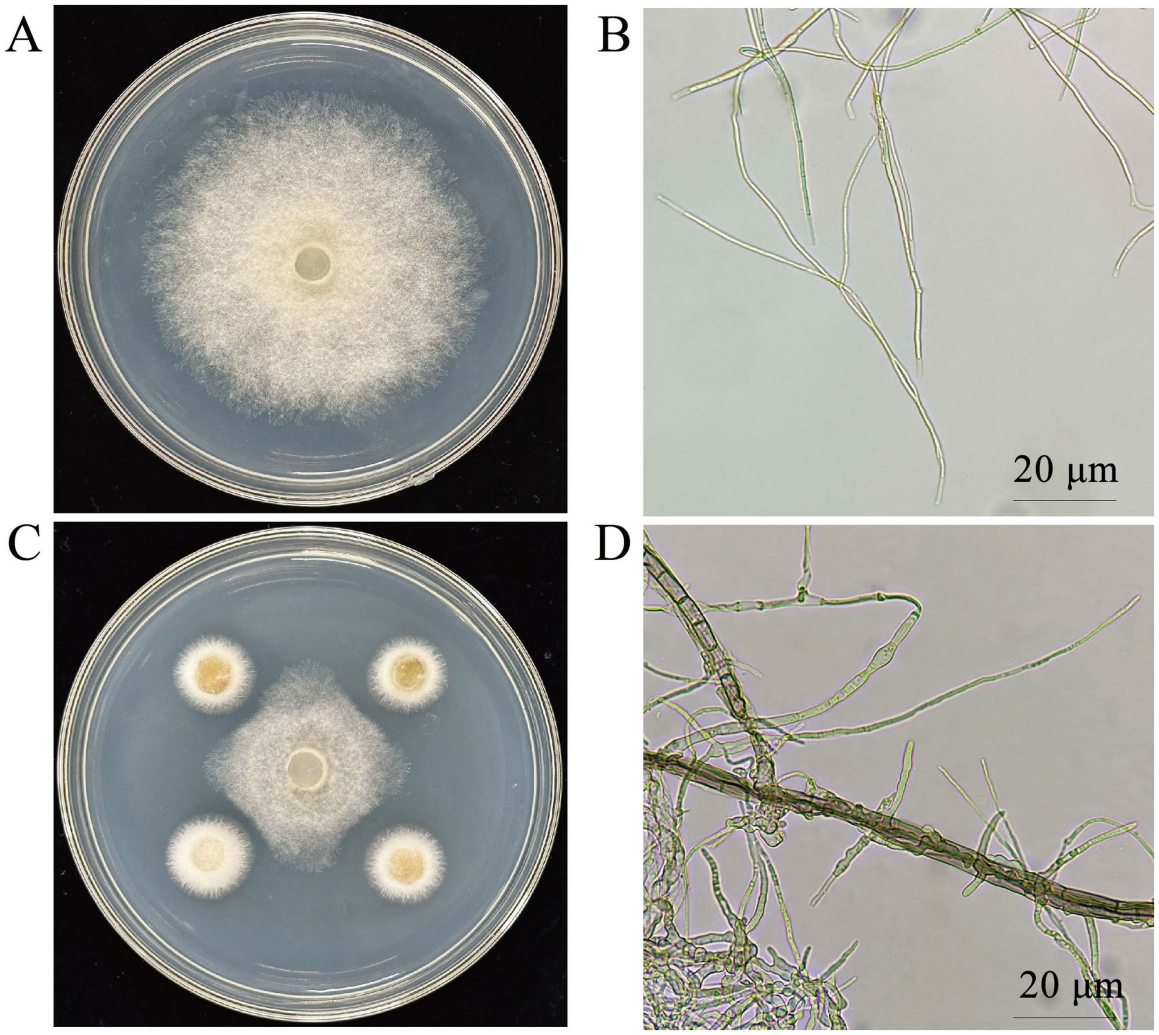
Effect of CY12 on the mycelium of *S. cucurbitacearum.* **(A)** Control colony; **(B)** Control hyphae; **(C)** Inhibition of the CY12 strain on *S. cucurbitacearum*; **(D)** Mycelia after inoculation with the CY12 strain.

Microscopic examination of the pathogen’s colonies and mycelia revealed that the untreated pathogen grew normally (Figure 1A), with uniform mycelial thickness and a smooth surface (Figure 1B). However, in the plate confrontation assay, fungal growth at the colony’s edge was notably suppressed (Figure 1C). The mycelia of the treated pathogen displayed distinct morphological alterations, including distortions, deformities, thickening, increased branching, and shortened length (Figure 1D).

### 3.2 Screening of the enzyme-producing abilities of antagonistic fungi

#### 3.2.1 Screening of amylase-producing strains

**Figure 2:**
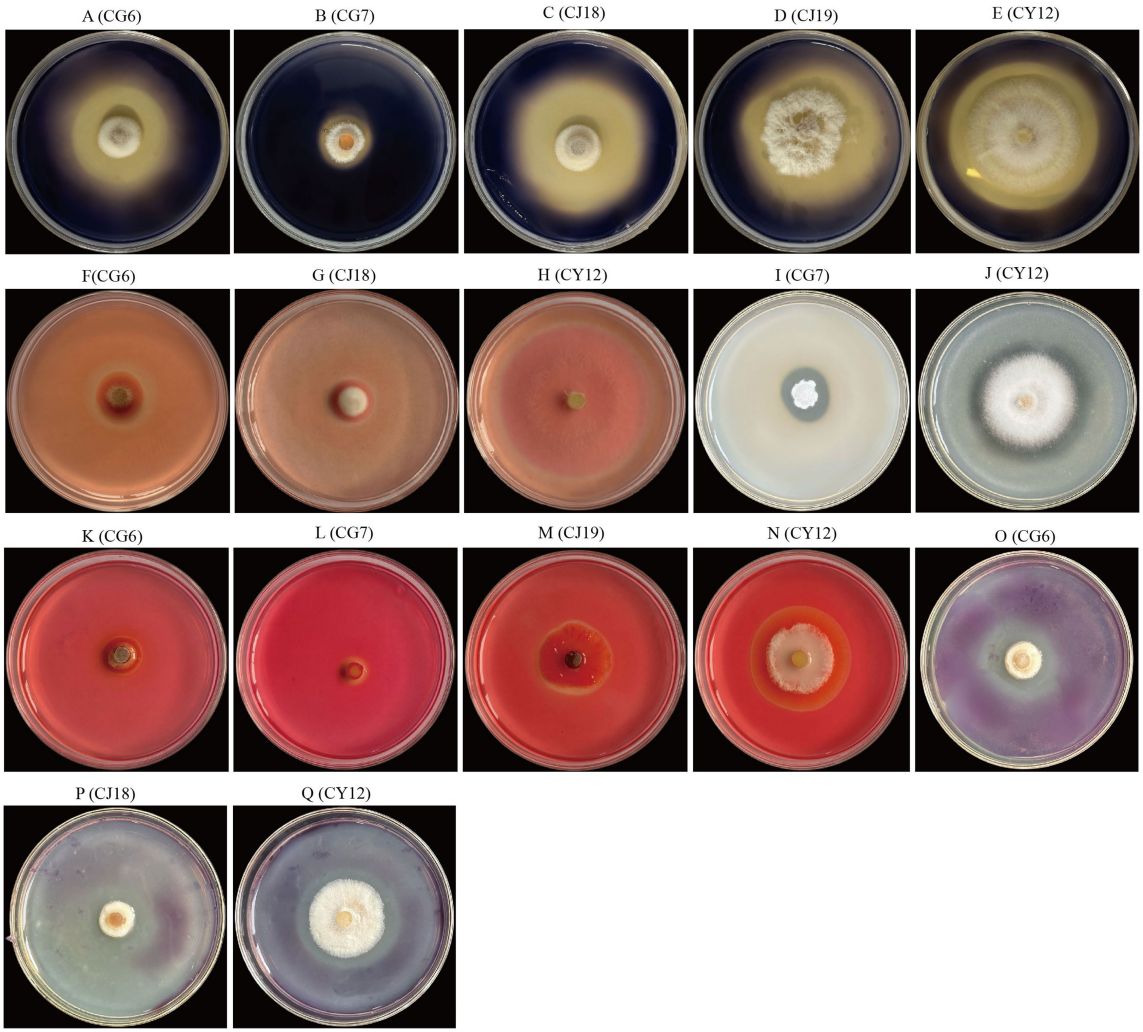
Enzyme production status of five strains of antagonistic fungi. **(A–E)** Amylase-producing strains; **(F–H)** *β*-glucanase-producing strains; **(I,J)** Protease-producing strains; **(K–N)** Cellulase-producing strains; **(O–Q)** Pectinase-producing strains.

The antagonistic strains CG6, CG7, CJ18, CJ19, and CY12 were tested for amylase production using a selective screening medium. Results showed that all five strains exhibited distinct decolorization zones, indicating amylase activity (Figures 2A–E).

#### 3.2.2 Screening of cellulase-producing strains

The five antagonistic fungi were inoculated onto cellulase screening medium, and four strains, CG6, CG7, CJ19, and CY12, formed clear hydrolysis zones, confirming their cellulase-producing ability (Figures 2K–N).

#### 3.2.3 Screening of pectinase-producing strains

Screening on a pectinase detection medium revealed that three strains, CG6, CJ18, and CY12, displayed pectinase-producing activity, as evidenced by the formation of hydrolysis zones (Figures 2O–Q).

#### 3.2.4 Screening of *β*-glucanase-producing strains

Plate assays for *β*-glucanase production indicated that three strains, CG6, CJ18, and CY12, produced visible transparent zones, confirming their *β*-glucanase activity (Figures 2F–H).

#### 3.2.5 Screening of protease-producing strains

Protease detection on selective medium identified two strains, CG7 and CY12, as protease producers, as they exhibited distinct transparent zones (Figures 2I,J).

Finally, by calculating the ratio of the diameter of the transparent circle or decolorization circle to that of the bacterial colony for the five strains, Table 2 was obtained. Judging from the above results, compared with the other four strains, CY12 has the abilities to produce amylase, cellulase, pectinase, *β*-glucanase, and protease simultaneously, demonstrating a relatively good ability to produce cell wall hydrolases, and it can be used for further experimental studies.

**Table 2:** The ability of five endophytic fungi to produce cell wall hydrolases.

Strain number	Amylase	*β*-Glucanase	Protease	Cellulase	Pectinase
CG6	1.87 ± 0.04b	1.25 ± 0.02c	0	1.09 ± 0.04b	2.23 ± 0.03a
CG7	1.45 ± 0.04c	0	1.74 ± 0.05a	1.52 ± 0.23a	0
CJ18	2.49 ± 0.15a	1.49 ± 0.04a	0	0	1.78 ± 0.05b
CJ19	1.49 ± 0.04c	0	0	1.08 ± 0.01b	0
CY12	1.21 ± 0.03d	1.31 ± 0.03b	1.22 ± 0.02b	1.15 ± 0.02b	1.78 ± 0.007b

Different letters after data indicate significant differences (*p* < 0.05).

### 3.3 Growth-promoting characteristics of antagonistic fungi

#### 3.3.1 Phosphate-solubilizing ability

**Figure 3:**
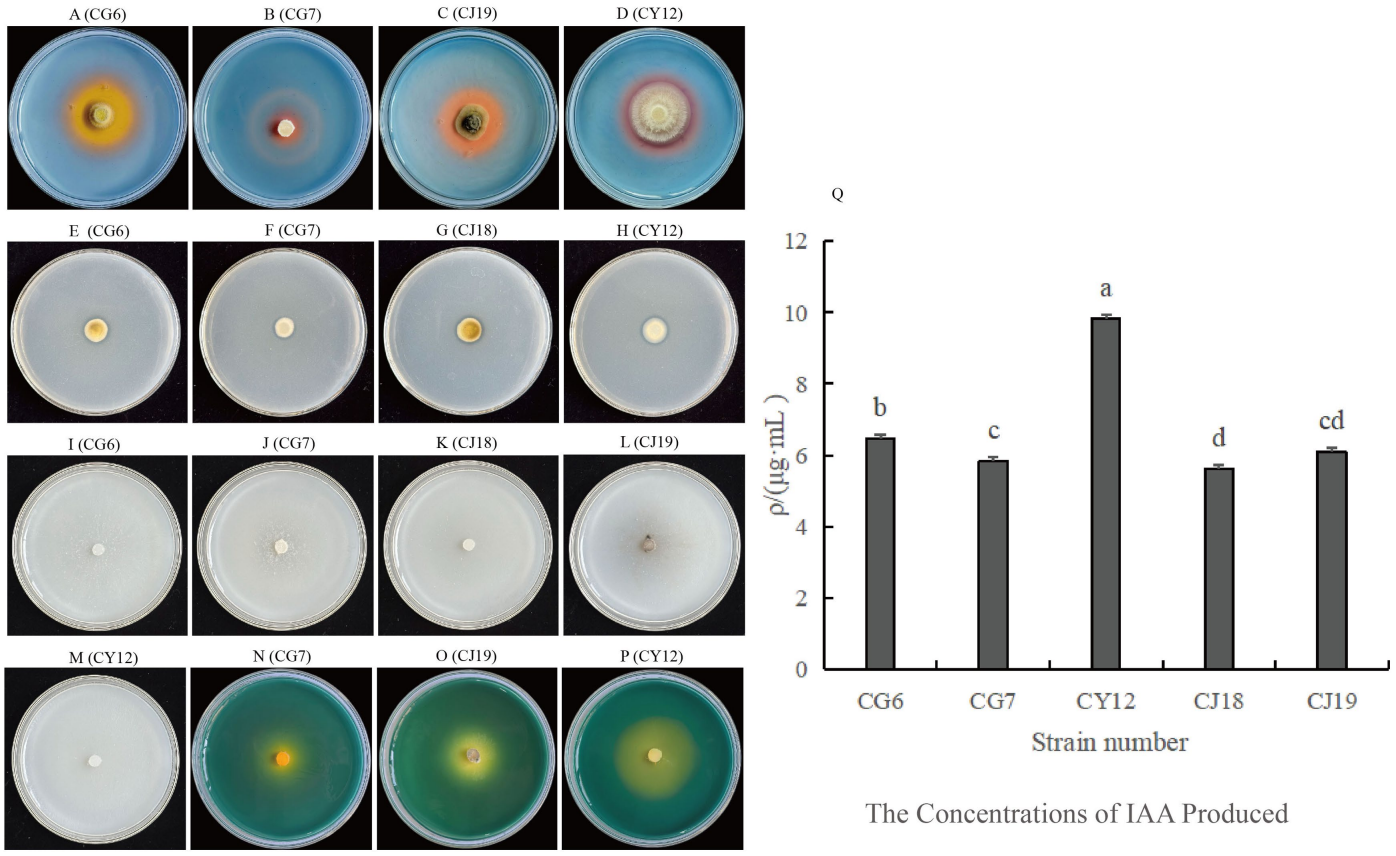
Growth-promoting abilities of five strains of antagonistic fungi. **(A–D)** Strains with siderophore-producing ability; **(E–H)** Strains with phosphate-solubilizing ability; **(I–M)** Strains with nitrogen-fixing ability; **(N–P)** Strains with potassium-solubilizing ability; **(Q)** Comparison of IAA-producing abilities among five strains.

Among the five dominant antagonistic strains, CG6, CG7, CJ18, CJ19, and CY12, four demonstrated phosphate-solubilizing activity. The *D1/d1* ratio, which represents phosphate-solubilizing efficiency, was calculated as follows: CG6 (1.20), CG7 (1.32), CJ18 (1.29), and CY12 (1.43) (Table 3; Figures 3E–H).

**Table 3:** The growth-promoting abilities of 5 antagonistic fungi.

Strain number	Phosphorus solubilization	Potassium releasing	nitrogen fixation	Siderophore-producing	IAA (mg/mL)
CG6	1.20 ± 0.02c	0	+	+	6.46 ± 0.14b
CG7	1.32 ± 0.06b	1.91 ± 0.04a	+	+	5.85 ± 0.33 cd
CJ18	1.29 ± 0.01b	0	+	-	5.63 ± 0.13d
CJ19	0	1.12 ± 0.03b	+	+	6.09 ± 0.05c
CY12	1.43 ± 0.03a	1.39 ± 0.01c	+	+	9.84 ± 0.10a

Different letters after data indicate significant differences (*p* < 0.05).

#### 3.3.2 Potassium-releasing ability

The potassium-releasing ability of the five strains was assessed, and three, CG7, CJ19, and CY12, exhibited positive results (Figures 3N–P). The *D2/d2* ratio for potassium-releasing ability was recorded as follows: CG7 (1.91), CJ19 (1.12), and CY12 (1.39) (Table 3).

#### 3.3.3 Siderophore-producing ability

The five antagonistic strains were inoculated onto CAS detection medium to assess siderophore production. After incubation, four strains, CG6, CG7, CJ19, and CY12, formed distinct orange-yellow halos around their colonies, indicating their ability to produce siderophores (Table 3; Figures 3A–D).

#### 3.3.4 Nitrogen-fixing ability

After five consecutive subcultures on nitrogen-free medium, all five screened strains continued to grow normally (Table 3; Figures 3I–M), confirming their nitrogen-fixing capability.

#### 3.3.5 IAA-producing ability

All five endophytic fungi demonstrated varying levels of IAA production. The IAA concentration was determined using the IAA standard curve (*y* = 0.0634x – 0.0255). Strains supplemented with tryptophan exhibited higher IAA secretion than those without. The IAA production levels for strains with tryptophan were as follows: CG6, 6.46 μg·mL^−1^; CG7, 5.85 μg·mL^−1^; CJ18, 5.63 μg·mL^−1^; CJ19, 6.09 μg·mL^−1^; and CY12, 9.84 μg·mL^−1^ (Table 3; Figure 3Q).

Based on the evaluation of enzyme production and growth-promoting functions, strain CY12 exhibited strong enzyme secretion and plant growth-promoting abilities. Therefore, CY12 was selected for further studies.

### 3.4 Molecular biology identification and morphological identification of strain CY12

#### 3.4.1 Morphological identification

**Figure 4:**
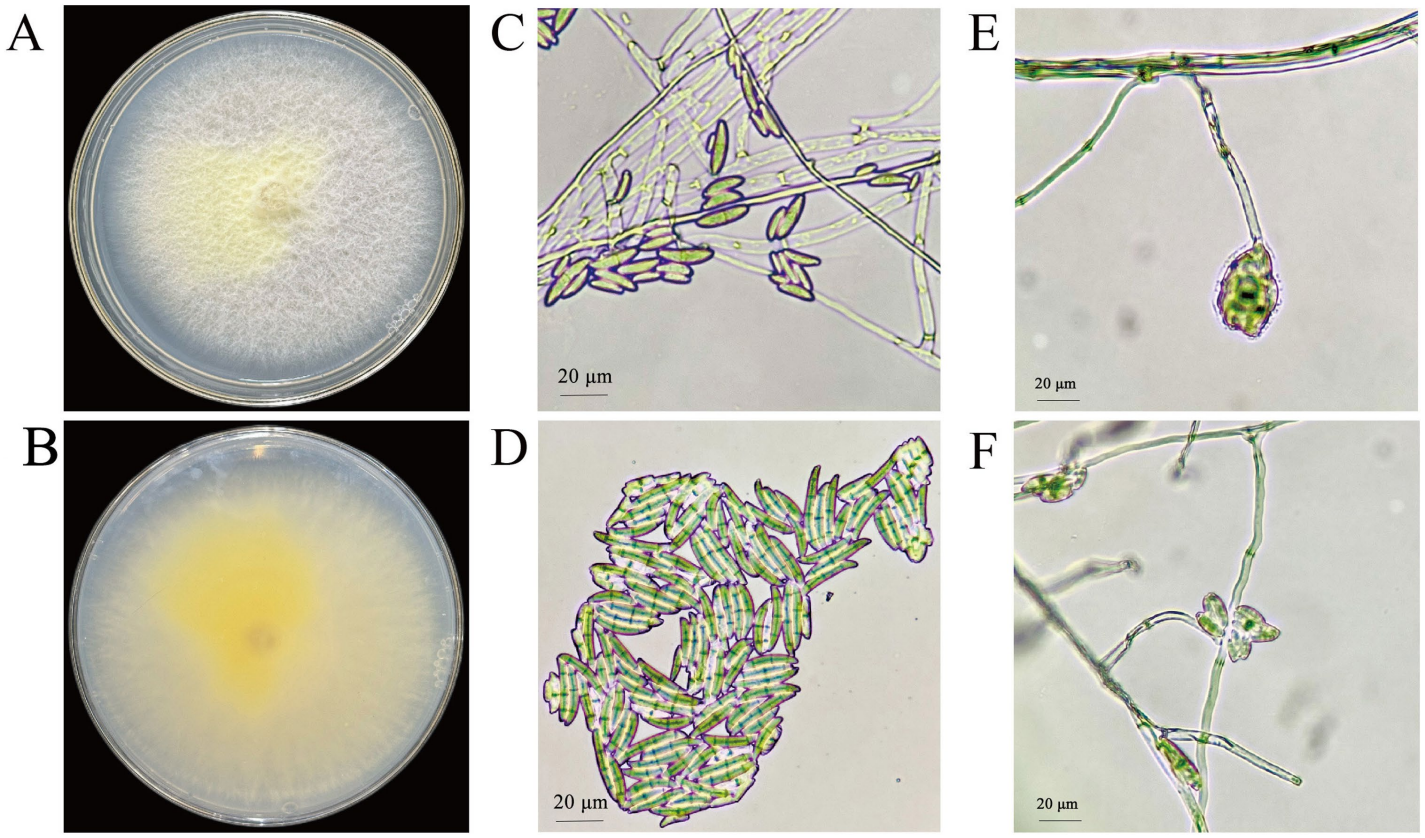
Colony and spore morphology of CY12. **(A,B)** Colony of CY12; **(C,D)** Conidia of CY12; **(E,F)** Conidium-producing structure.

On PDA medium, the colonies of strain CY12 displayed a white-to-yellow coloration with a sparse, villous texture (Figure 4). The strain produced light yellow or purple-pink metabolites. The reverse side of the colony was yellow. Microscopic examination revealed sickle-shaped spores, with spiny top cells that curved inward.

#### 3.4.2 Molecular biology identification

**Figure 5:**
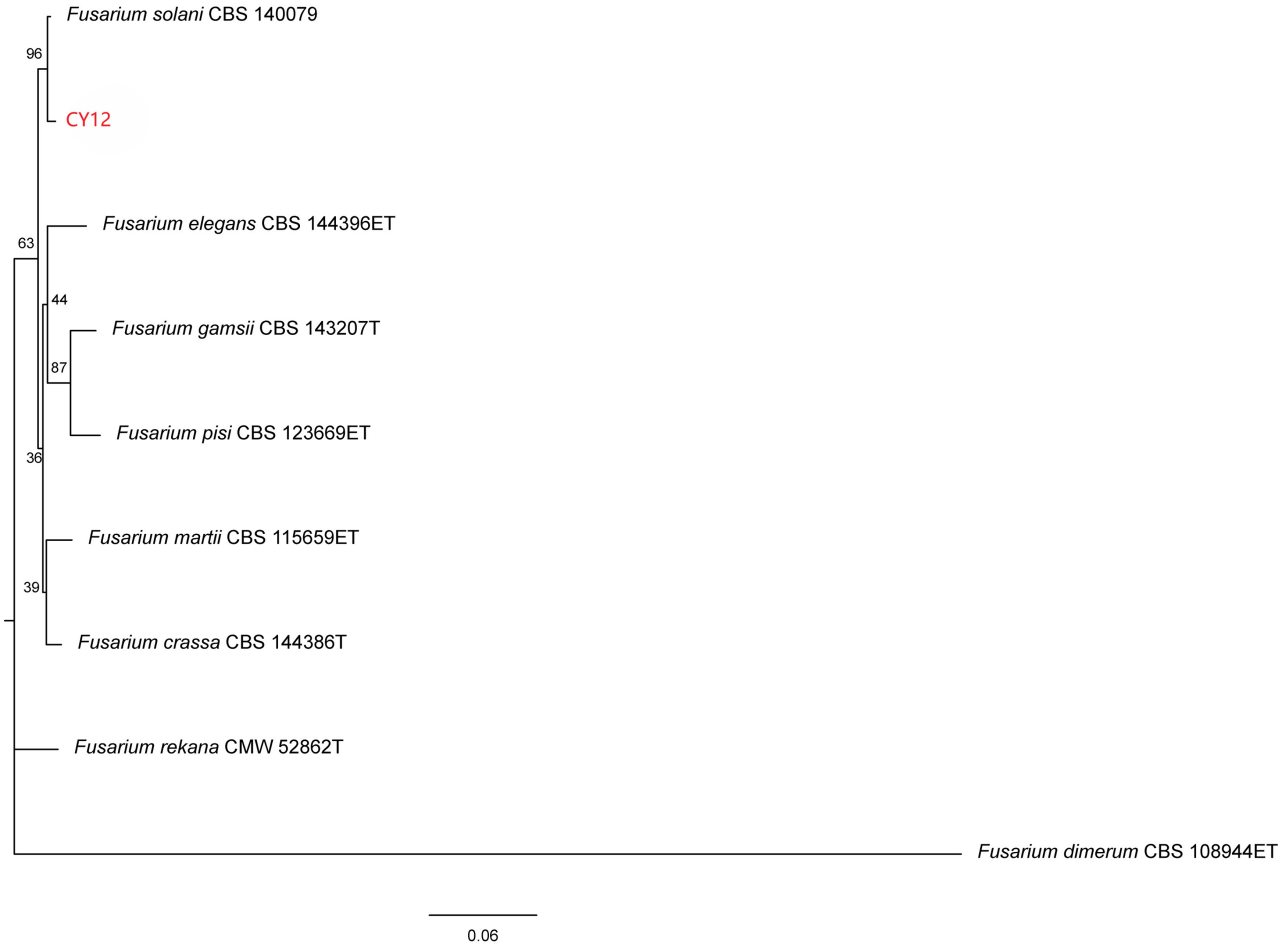
Neighbor-joining phylogenetic tree of the antagonistic fungus CY12 was constructed by MEGA 11.0.

The ITS, TEF, and RBP gene sequences of strain CY12 were analyzed using the NCBI database. A phylogenetic tree was constructed using MEGA 11.0 software based on ITS, TEF, and RBP2 sequences through the maximum likelihood method (Figure 5). The results indicated that strain CY12 had the closest genetic relationship to *Fusarium solani*, clustering within the same branch with over 96% sequence similarity.

In summary, based on morphological and molecular analyses, strain CY12 was identified as *Fusarium solani*.

### 3.5 Determination of the antifungal spectrum of strain CY12

Strain CY12 demonstrated varying degrees of inhibitory activity against six plant pathogenic fungi. The strongest inhibition was observed against the leaf spot pathogen of Rhododendron, with an inhibition rate of 71.41%, while the weakest effect was recorded against the leaf spot pathogen of *Hibiscus mutabilis*, with an inhibition rate of 36.76% (Table 4).

**Table 4:** The impact of antagonistic fungus CY12 on different pathogenic fungi.

Pathogenic fungi	Inhibition (%)	Pathogenic fungi	Inhibition (%)
Impatiens wilt disease (*Fusarium oxysporum*)	58.73 ± 0.81c	Ivy leaf blight (*Apiospora intestini*)	65.16 ± 1.92b
Rhododendron leaf spot (Pestalotiopsis)	71.41 ± 1.17a	Blueberry gray mold (*Botrytis cinerea*)	38.40 ± 1.01e
*Hibiscus mutabilis* leaf spot (*Fusarium sambucinum*)	36.76 ± 4.34e	Hydrangea leaf spot (*Epicoccum sorghinum*)	52.99 ± 2.10d

Different letters after data indicate significant differences (*p* < 0.05).

These findings suggest that strain CY12 possesses broad-spectrum antifungal activity.

### 3.6 *In vivo* control effect of CY12 against leaf spot disease of *Impatiens hawkeri*

Using the spore suspension inoculation method, a 7-day treatment resulted in distinct differences between the two branches subjected to different treatments on the same plant. On the branch inoculated only with the pathogen spore suspension, black-brown lesions developed at the wounded sites on the leaves after 7 days. Some lesions exceeded 2 cm^2^ in size, and in severe cases, leaf necrosis and petiole detachment were observed.

**Figure 6:**
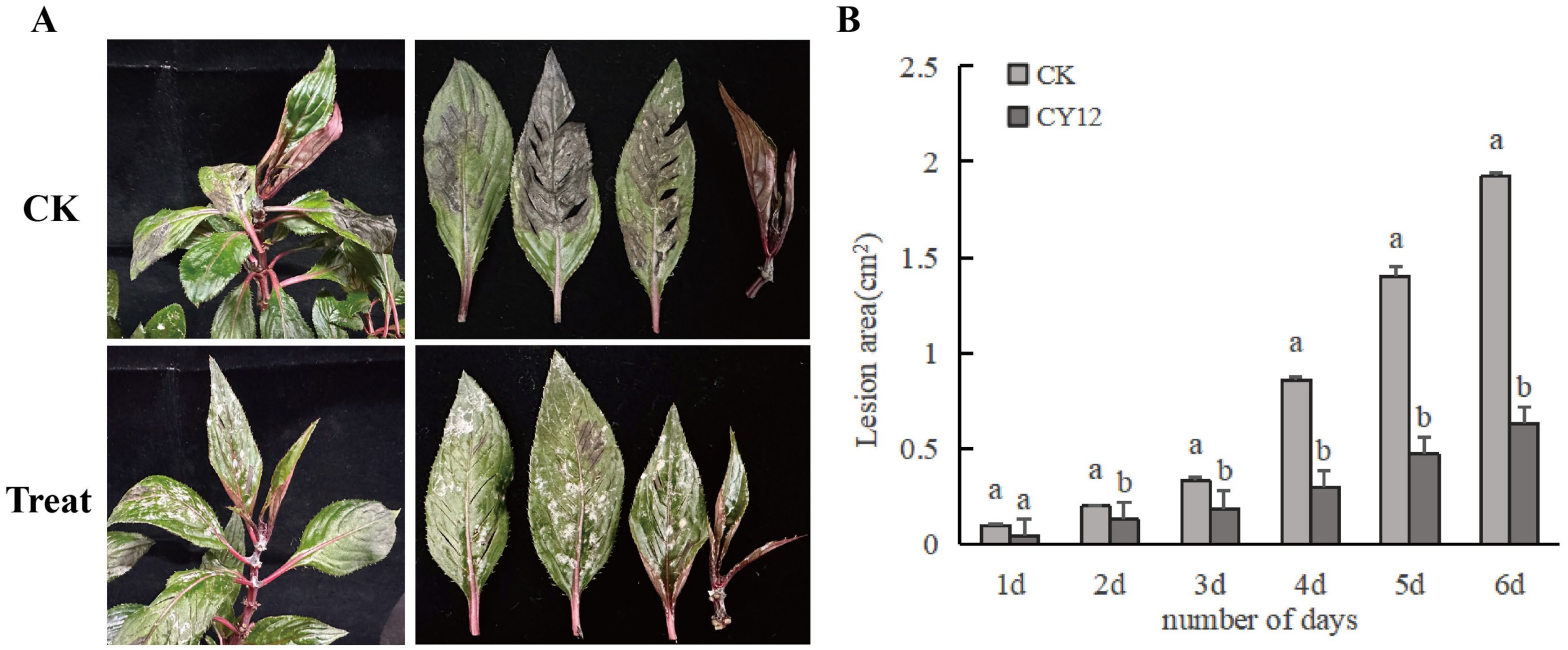
*In vivo* control effect of CY12 against leaf spot disease of *Impatiens hawkeri*. **(A)** *In vivo* inhibitory effect of CY12 on pathogenic fungi after 7 days; **(B)** Lesion changes in the control group and the experimental group within 7 days.

In contrast, on the branch initially inoculated with the pathogen spore suspension and subsequently treated with the CY12 fermentation broth 4 h later, disease symptoms were significantly reduced. Apart from some residual mycelium from the biocontrol fermentation broth, only a few leaves exhibited lesions, with a lesion area of just 0.63 cm^2^ (Figure 6). The diseased area was substantially smaller than that observed on leaves inoculated solely with the pathogen fermentation broth.

### 3.7 Growth-promoting effect of CY12 on *Impatiens hawkeri* seeds

In the seed germination experiment, both the control and CY12-treated *I. hawkeri* seeds began to germinate within 24 h. The control group reached its maximum germination count at 48 h. In comparison, the CY12-treated seeds exhibited an accelerated germination rate, with a sharp increase in the number of germinated seeds. By 24 h, the CY12-treated seeds had already reached their maximum germination count, and the root lengths of the germinated seeds in the CY12 group were significantly longer than those in the control group (Table 5).

**Table 5:** The effects of CY12 on the seed germination and seedling growth of *Impatiens hawkeri.*

Treatment	Plant height (cm)	Germination time (h)	Root length (cm)	Sending length (cm)	Root length/Sending length (%)	Germination rate (%)
Control	13.09 ± 0.80a	24	6.62 ± 0.76a	5.23 ± 0.35a	1.26 ± 0.08a	98.33 ± 1.51a
CY12	16.55 ± 0.46b	24	8.65 ± 0.46b	5.71 ± 0.29b	1.52 ± 0.03b	93.00 ± 2.10b

Different letters after data in the same column indicate significant differences among treatments (*p* < 0.05).

**Table 6:** Culture media and drugs.

Culture media	Drugs
Potato dextrose agar medium	Potato 200 g, Anhydrous dextrose 20 g, Agar 20 g, H_2_O 1,000 mL
Potato dextrose medium	Potato 200 g, Anhydrous dextrose 20 g, H_2_O 1,000 mL
Czapek medium	Sucrose 30 g, Agar 20 g, KCl 0.5 g, NaNO_3_ 2 g, K₂HPO₄ 1 g, FeSO₄ 0.01 g, MgSO₄·7H₂O 0.5 g, H_2_O 1,000 mL
Organic phosphorus medium	Anhydrous dextrose 10 g, Lecithin 5 g, Agar 20 g, (NH₄)₂SO₄ 0.5 g, NaCl 0.3 g, K₂SO₄ 0.3 g, MgSO₄·7H₂O 0.3 g, MnSO₄ 0.03 g, FeSO₄ 0.03 g, H_2_O 1,000 mL
Nitrogen-free medium	Mannitol 10 g, Agar 20 g, KH₂PO₄ 0.2 g, NaCl 0.2 g, CaSO₄ 0.1 g, CaCO₃ 5 g, MgSO₄·7H₂O 0.2 g, H_2_O 1,000 mL
Siderophore detection medium	Sucrose 10 g, Peptone 5 g, Yeast Extract 1 g, Agar 20 g, KH₂PO₄ 0.2 g, MgSO₄·7H₂O 0.2 g, H_2_O 1,000 mL
Aleksandrov medium	Sucrose 5 g, Agar 20 g, Bromothymol Blue 0.1 g, Potassium Feldspar Powder 1 g, Na₂HPO₄ 2 g, MgSO₄·7H₂O 0.5 g, CaCO₃ 0.1 g, FeCl₃ 0.05 g, H_2_O 1,000 mL
Amylase detection medium	Soluble Starch 1 g, Agar 2 g, K₂HPO₄ 0.03 g, KNO₃ 0.1 g, NaCl 0.05 g, MgSO₄·7H₂O 0.1 g, H_2_O 1,000 mL
Protease detection medium	Skim Milk Powder 5 g, Agar 20 g, Peptone 10 g, Beef Extract 3 g, NaCl 5 g, H_2_O 1,000 mL
Cellulase detection medium	Peptone 5 g, Agar 20 g, Congo Red 0.05 g, CMC-Na 1 g, KH₂PO₄ 1 g, MgSO₄·7H₂O 0.5 g, (NH₄)₂SO₄ 1 g, H_2_O 1,000 mL
Pectinase detection medium	Pectin 10 g, Potassium Feldspar Powder 0.5 g, Anhydrous dextrose 1 g, Agar 20 g, Na₂SO₄ 1 g, KH₂PO₄ 1 g, KCl 1 g, MgSO₄·7H₂O 0.5 g, H_2_O 1,000 mL
*β*-Glucanase detection medium	Dextran 5 g, Congo Red 0.05 g, Agar 20 g, KCl 0.5 g, K₂HPO₄ 1 g, MgSO₄·7H₂O 0.5 g, NaNO₃ 2 g, H_2_O 1,000 mL

**Figure 7:**
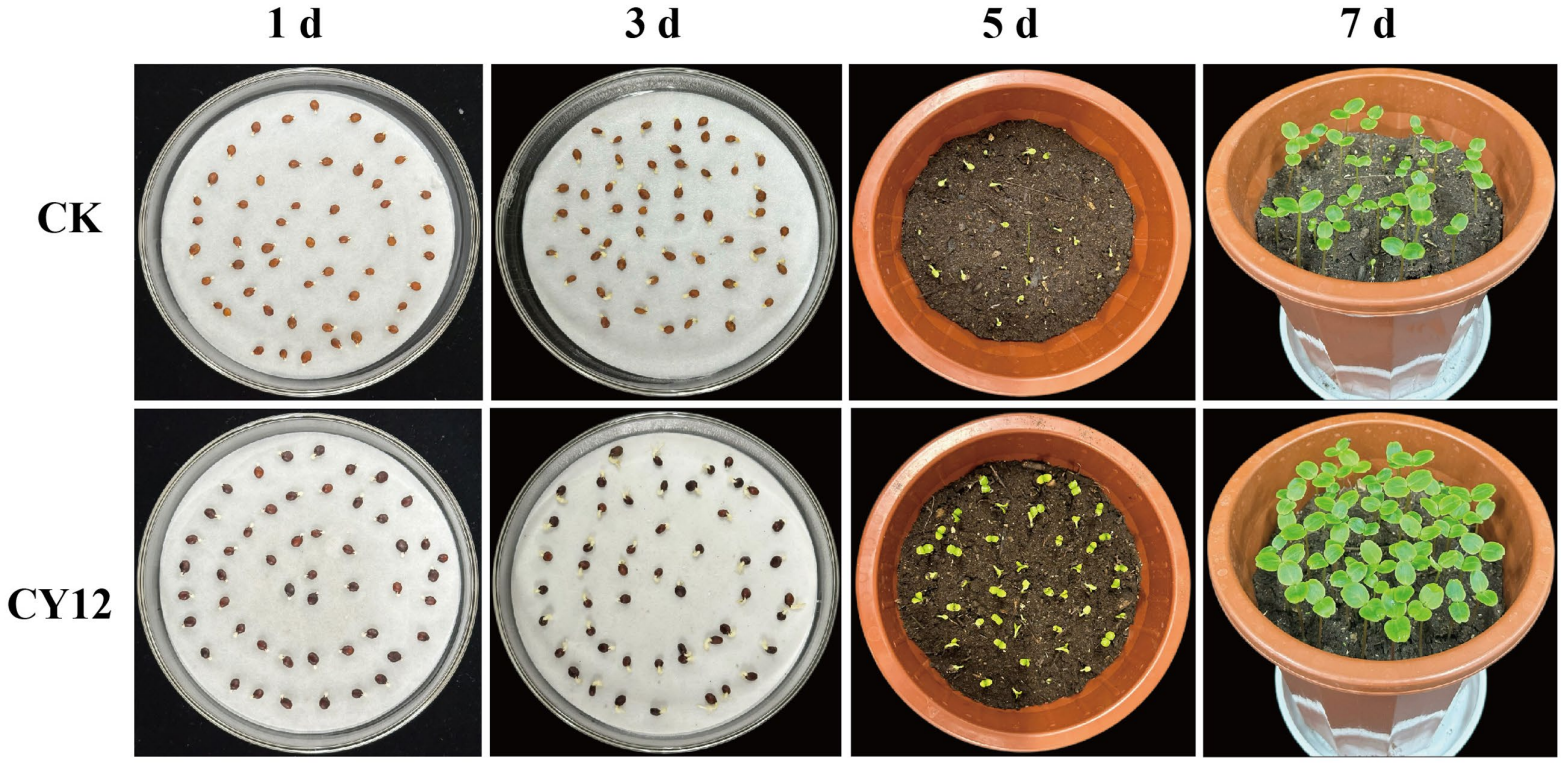
Promoting effect of CY12 on the seed germination of *Impatiens hawkeri*.

After transplanting the seedlings into flowerpots, clear differences in growth became apparent. By day 5, some seedlings in the control group had emerged from the soil, showing green leaves and stems, while others remained buried, leading to uneven growth. In contrast, nearly all CY12-treated seeds had successfully emerged, revealing green stems and new leaves. Only a few seedlings had yet to push their cotyledons through the soil, and overall, seedling growth appeared more uniform. By day 7, all seedlings in the control group had emerged and continued growing, but noticeable growth disparities remained. Meanwhile, seedlings in the CY12-treated group exhibited more vigorous growth, with new leaves of similar size and uniform plant heights (Figure 7).

Measurements of randomly selected seedlings from each treatment group revealed significant differences in root length, bud length, and plant height (*p* < 0.05) (Table 5).

These results suggest that strain CY12 promotes both seed germination and seedling growth in *I. hawkeri*.

## 4 Discussion

*Fusarium solani* belongs to the genus *Fusarium*, one of the most diverse groups of endophytic fungi, comprising approximately 70 species (Torres-Cruz et al., 2022). While commonly recognized as a soil saprophyte with the potential to cause severe plant diseases, many *Fusarium* species have also been reported to establish asymptomatic infections in various plant species. *F. solani*, in particular, is widely distributed as an endophytic fungus in numerous host plants. Previous studies have demonstrated the presence of endophytic *F. solani* in the roots of various grasses, including *Holcus lanatus*, which thrives in wet and waterlogged environments (Sánchez Márquez et al., 2010), as well as *Ocimum* (Adi One et al., 2022) and *Dendrobium falconeri Hook*. f. (Shah et al., 2019). Beyond gramineous plants, *F. solani* has also been isolated from the roots of several leguminous species (Moricca and Ragazzi, 2008), while endophytic *Fusarium oxysporum* isolates have been identified in the roots of various vegetable crops (Kim et al., 2007). Additionally, *F. solani* has been detected in the root systems of mangroves (Xing and Guo, 2011). These findings indicate that *F. solani* is a widespread fungal endophyte capable of colonizing the roots of grasses and other plants without causing disease.

Research has shown that certain non-pathogenic *Fusarium* species can act as effective biocontrol agents. Elmer (2004) found that the non-pathogenic *F. oxysporum* CS-20 exhibited strong inhibitory effects against asparagus crown rot. Similarly, non-pathogenic *F. oxysporum* strains have been reported to suppress plant diseases in crops such as tomato (Fuchs et al., 1999; Shishido et al., 2005) and cucumber (Abeysinghe, 2006; Mandeel and Baker, 1991.

In the present study, strain CY12, identified as *F. solani*, did not cause disease in *I. hawkeri*. Instead, it exhibited strong antagonistic activity against the pathogen responsible for *I. hawkeri* leaf spot disease, along with six other plant pathogens. Furthermore, through growth-promoting function assessments and seed germination experiments, CY12 was confirmed to promote *I. hawkeri* seed germination and seedling growth. These findings further support the notion that some fungal strains, traditionally regarded as plant pathogens, can colonize host plants without causing disease, while simultaneously improving plant resistance to pathogens and promoting plant growth.

The inhibitory effects of antagonistic fungi on pathogenic fungi primarily involve several mechanisms. These include competition for space and nutrients, where antagonistic fungi outcompete pathogens for living areas (Solanki et al., 2014), and direct antagonistic interactions, which lead to morphological abnormalities in pathogen mycelia, such as increased branching, apical swelling (Sun et al., 2023), and cytoplasmic exudation (Li et al., 2025). Additionally, antagonistic fungi inhibit conidial germination and restrict mycelial growth (Chen et al., 2008; Nian et al., 2021). The antagonistic effect is also manifested in the production of cell wall-degrading enzymes to inhibit the growth of pathogenic fungi. Another mechanism is hyperparasitism, where antagonistic fungi establish direct contact with pathogens, wrapping around, penetrating, and parasitizing their mycelia (Wang X. et al., 2024). Similarly, strain CY12 demonstrated rapid growth and effectively occupied available space in the confrontation assay. This reflects the competitive effect of CY12 on pathogenic fungi. It formed a distinct inhibition zone against the pathogen, significantly suppressing the mycelial growth of *Botrytis cinerea* and inducing structural deformities such as mycelial thickening, shortening, and excessive branching. Additionally, strain CY12 exhibited the capacity to produce multiple cell wall-degrading enzymes, which likely contributed to its ability to weaken and degrade pathogen structures. This reflects the antagonistic and hyperparasitic effects of CY12 on pathogenic fungi.

Beyond their biocontrol capabilities, many antagonistic fungi also exhibit strong growth-promoting effects (Khan et al., 2020; Zhou et al., 2021). Within the genus *Fusarium*, *F. oxysporum* GW, isolated from wheat, has been reported to promote wheat growth while also suppressing the growth of the weed *Avena fatua* (Asim et al., 2022). Similarly, endophytic *F. oxysporum* isolated from *Cotoneaster multiflorus* has been found to facilitate phosphate solubilization, potassium release, and IAA production (Lü et al., 2023). The results of this study suggest that strain CY12 similarly promotes seed germination, seedling development, and root elongation in *I. hawkeri*. Other antagonistic fungi, such as *Paraphaosphaeria* sp. JRF11, isolated from *Carya illinoinensis*, have also been shown to solubilize phosphorus, release potassium, and produce IAA, leading to increased tomato seedling growth (Shan et al., 2024). Likewise, strain CY12 not only exhibited phosphate-solubilizing, potassium-releasing, and IAA-producing abilities but also demonstrated nitrogen-fixing and siderophore-producing capacities. These combined traits are likely key factors contributing to its growth-promoting effects on plants. To further verify the growth-promoting potential of strains with multiple plant-beneficial functions, pot experiments are commonly employed. In this study, a seed germination experiment using seed soaking with CY12 fermentation broth confirmed that strain CY12 significantly increased seed germination as well as root and bud growth in *I. hawkeri*.

This study successfully isolated endophytic fungi from the tissues of *Q. spinosa* and evaluated their antifungal activities against the pathogen responsible for *I. hawkeri* leaf spot disease. Among the isolates, strain CY12 was identified as an outstanding candidate due to its broad-spectrum and high antifungal activity. The *in vivo* control effect of CY12 on *I. hawkeri* leaf spot disease was assessed, and its growth-promoting functions were verified.

In the plate experiment, we observed that the endophytic fungi showed obvious inhibitory zones against *S. cucurbitacearum*, which could inhibit the growth of its hyphae. The antifungal rate reached 68.13%, indicating that the endophytic fungi can effectively inhibit the growth of pathogenic fungi *in vitro*. Meanwhile, in the pot experiment for disease prevention, the area of disease spots on the potted plants treated with CY12 was significantly smaller than that of the control group. This further proves that CY12 has a preventive and control effect against pathogenic fungi under the pot conditions simulating the natural environment.

Although there is a lack of field trial data, based on the favorable results of the plate experiment and the pot experiment, we believe that CY12 has the potential to serve as a biocontrol agent against *S. cucurbitacearum.*

In conclusion, strain CY12 demonstrated strong inhibitory effects against *S. cucurbitacearum*, effectively reducing the occurrence of *I. hawkeri* leaf spot disease. These findings suggest that CY12 represents a promising microbial resource for the biological control of *I. hawkeri* leaf spot disease. First, as a biocontrol agent, experimental results demonstrate that CY12 inhibits the growth of multiple plant pathogens and reduces the incidence of *S. cucurbitacearum* induced leaf spot disease in *I. hawkeri*. Second, CY12 promotes plant growth through multiple mechanisms: it produces indole-3-acetic acid (IAA), solubilizes phosphorus and potassium, synthesizes siderophores, and modulates phytohormone levels to enhance nutrient availability.

In future research, field trials can be conducted to verify the stability of the endophytic fungi’s effect in inhibiting pathogenic fungi. Additionally, the functions of endophytic antagonistic fungi in inducing plant resistance and the analysis of their metabolites can continue to be explored.

## Data Availability

All sequences of the strain have been uploaded to NCBI and the accession number is PQ849072 (ITS), PQ855391 (TEF), PQ855392 (RBP2).
